# Upregulation of lncRNA NR_046683 Serves as a Prognostic Biomarker and Potential Drug Target for Multiple Myeloma

**DOI:** 10.3389/fphar.2019.00045

**Published:** 2019-01-31

**Authors:** Hang Dong, Siyi Jiang, Yunfeng Fu, Yanwei Luo, Rong Gui, Jing Liu

**Affiliations:** ^1^Department of Blood Transfusion, The Third Xiangya Hospital of Central South University, Changsha, China; ^2^Department of Hematology, The Third Xiangya Hospital of Central South University, Changsha, China

**Keywords:** lncRNA, NR_046683, multiple myeloma, prognostic factor, drug target

## Abstract

**Aim:** To investigate the prognostic value of lncRNA NR_046683 in multiple myeloma (MM).

**Methods:** High-throughput lncRNA array was combined with bioinformatics techniques to screen differentially expressed lncRNA in MM. qRT-PCR was adopted to determine the expression of target lncRNAs in MM patients and controls.

**Results:** It was found for the first time that lncRNA NR_046683 is closely related to the prognosis of MM. It was also detected in tumor cell lines KM3, U266, especially in drug-resistant cell lines KM3/BTZ and MM1R. The NR_046683 expression differed significantly in patients of different MM subtypes and staging. Moreover, the overexpression of NR-046683 is closely related to β_2_-microglobulin. We also found that the overexpression of NR-046683 correlates to chromosomal aberrations, such as del(13q14), gain 1q21, and t(4;14).

**Conclusion:** lncRNA NR_046683 can serve as a novel biomarker for potential drug target and prognostic prediction in MM.

## Introduction

Multiple myeloma (MM) is a hematologic malignancy caused by the proliferation of plasma cells in bone marrows. MM is the second most common cancer of blood system after non-Hodgkin lymphoma, and is associated with the signs and symptoms of bone pain, pathologic fractures, hypercalcemia, anemia, and renal failure (Chen W. C. et al., 2017; Cowan et al., 2018). MM usually develops from monoclonal gammopathy of under determined significance (MGUS) (Weiss et al., 2009; Kyle and Rajkumar, 2010). The development from MGUS to MM is accompanied by genetic variations such as cytogenetic aberrations, primary or secondary chromosomal translocation and oncogene activation. Understanding these genetic variations is of high importance for prognostic and response prediction.

Non-coding RNAs (ncRNAs) are defined as RNA molecules that do not encode proteins, but recent evidence has proven that peptides/proteins encoded by ncRNAs do indeed exist and may have an important role in regulating tumor energy metabolism, epithelial to mesenchymal transition of cancer cells (Zhu et al., 2018). These peptides/proteins represent promising drug targets for fighting against tumor growth or biomarkers for predicting the prognosis of cancer patients. Depending on length, ncRNAs are divided into short and long ncRNAs (lncRNAs). lncRNAs are usually longer than 200 nt and highly conservative during mammal evolution (including human). lncRNAs are involved in many biological processes, such as gene transcription regulation, maintenance of genomic integrity, X-chromosome inactivation, genomic imprinting, cell differentiation and development. lncRNAs are known to be abnormally expressed in cancer tissues and involved in carcinogenesis or tumor suppression (Poliseno et al., 2010; Hung and Chang, 2014). The human lncRNA catalog has been constantly expanding in recent years. The largest database of lncRNAs transcripts contains over 90,000 human lncRNA genes (e.g., 51, 382 LNCipedia v5.0; 96, 308 Noncode v5.0) (Volders et al., 2015; Fang et al., 2018). Although, the working mechanism of most lncRNAs remains unclear, the dysregulation of different lncRNAs contributes to the development and metastasis of different tumors, such as breast cancer, gastric cancer, hematologic cancer, and lung cancer (Chen J. S. et al., 2016; Rodriguez-Malave and Rao, 2016; Alvarez-Dominguez and Lodish, 2017; Cai et al., 2017; Fu et al., 2018).

The latest bioinformatics technique was used in combination with high-throughput lncRNA database of a small sample size to identify lncRNAs. These lncRNAs influenced the therapeutic response and efficacy of MM. Real-time quantitative polymerase chain reaction (qRT-PCR) was adopted to determine the expression of target lncRNAs in plasma cells from the bone marrows of MM patients and controls. lncRNA-mRNA co-expression network was established by combining with medical history of patients. It was then analyzed whether or not the expression of target lncRNAs could be used to predict the prognosis of MM. This was of high clinical significance to identify effective biomarkers and new treatment targets for MM.

## Materials and Methods

### Clinical Data

From January 2012 to January 2018, 86 cases (53 males and 33 females) with MM treated at the Third Xiangya Hospital of Central South University were recruited. Their bone marrow samples and clinical data were collected. The median age of MM onset was 55 years old (44–78 years). All included cases had complete clinical and pathological data (Table 1). All MM cases were diagnosed according to the diagnostic criteria developed by International Myeloma Working Group (IMWG) (Rajkumar et al., 2011). Given the lack of bone marrow samples from normal donors, the sample variation was reduced by selecting 30 cases with iron deficiency anemia (IDA) as controls, and their bone marrow samples were collected. The sample collection was approved by the hospital’s ethics committee (approval number: 2016121) and the informed consents were signed by all cases.

**Table 1:** Characteristics of study population.

Clinical characteristics	MM patients	Value
Sex		
	Male	53 (62%)
	Female	33 (38%)
Age (yr)		Median 55 (range: 44–78)
International staging system		
	Stage 1	16 (19%)
	Stage 2	33 (38%)
	Stage 3	37 (43%)
Durie-Salmon stage		
	Stage 1	40 (47%)
	Stage 2	20 (23%)
	Stage 3	26 (30%)
Isotype		
	IgG	43 (50%)
	IgA	22 (26%)
	Light chain	12 (14%)
	Unclassified	9 (10%)
Percentage of myeloma cells in BM		
	<40%	57 (66%)
	≥ 40%	29 (34%)
Bone disease		
	No	7 (8%)
	Yes	79 (92%)
Renal insufficiency		
	No	67 (78%)
	Yes	19 (22%)
Cytogenetic abnormality		
	No	29 (34%)
	Yes	57 (66%)
Hemoglobin (g/dl)		101.18 ± 26.11
Platelet count (×10^9^/L)		169.29 ± 88.43
Neutrophil (×10^9^/L)		2.81 ± 1.96
Albumin (g/L)		34.21 ± 6.46
Globulin (g/L)		32.90 ± 22.40
LDH (IU/L)		265.23 ± 161.23
β_2_-MG (μg/ml)		5.35 ± 3.88
Creatinine (μmol/L)		106.18 ± 122.22
Serum Calcium (mmol/L)		2.21 ± 0.20


*MM, multiple myeloma; BM, bone marrow; LDH, lactate dehydrogenase; β_2_-MG, β_2_-microglobulin.*

### RNA Extraction

Total RNA extraction was performed from bone marrow samples of MM and IDA patients. Prior to use, RNA samples were stored at -80°C. NanoDrop ND-1000 was used to determine RNA concentration and activity. RNA integrity was assessed by denaturing gel electrophoresis.

### Result Analysis of High-Throughput lncRNA Array and Differential Expression

Labeling and array hybridization were performed suing Agilent One-Color Microarray-Based Gene Expression Protocol (Agilent Technology). rRNA was removed from total RNA using mRNA-ONLY TM, Eukaryotic mRNA Isolation Kit (Epicenter). Each sample was amplified and transcribed into fluorescent-labeled cRNA. The labeled cRNAs were purified using RNeasy Mini Kit (Qiagen), and NanoDrop ND-1000 was used to determine its concentration and activity. Microarray hybridization was performed (Arraystar Human LncRNA Array V4.0). Microarray images were generated using Agilent Feature Extraction (v11.0.1.1), and the original data were read. Quantile normalization was performed on the original data using GeneSpring GX v12.1 (Agilent Technologies) with data processing. Differentially expressed lncRNA were identified based on fold change and *p* value. The fold-change threshold for upregulated and downregulated genes was >2.0 with *p* < 0.05.

### Verification by Quantitative RT-PCR

RNA was reversely transcribed into cDNA using SuperScript III Reverse Transcriptase (Invitrogen, Grand Island, NY, United States). qRT-PCR (Arraystar) was performed using ViiA 7 Real-time PCR System (Applied Biosystems) and 2 × PCR Master Mix. The reaction conditions were as follows: incubation at 95°C for 10 min, 95°C 10 s and 60°C 1 min, a total of 40 cycles. β-actin was taken as internal reference and the expression of target lncRNAs was normalized based on β-actin. ΔCt value indicated the lncRNA expression level. Primers for each gene are shown in Table 2.

**Table 2:** Primers designed for qRT-PCR validation of candidate lncRNAs.

	Primer	Tm (°C)
β-actin	F:5′ GTGGCCGAGGACTTTGATTG3′ R:5′ CCTGTAACAACGCATCTCATATT 3′	60
NR_104063	F:5′ AAGCAAAAGTGCAGAAAACCAT 3′ R:5′ CTGAGTGACCTGTTGCCTGAA 3′	60
T283430	F:5′ AGAAATGGGATACCAAAGGAGT 3′ R:5′ TCCTCTCTATCCTTCAGCACAT 3′	60
NR_046683	F:5′ GATGTGATGCCTGAAGATGTG 3′ R:5′ TTCTGTGCTGCCAGTTGTG 3′	60
uc021pbg.1	F:5′ CTACCTGAGCCAGTTCTCCTAA 3′ R:5′ GGGTTCCTCATCGGTGTAAT 3′	60


### Functional Analysis of lncRNA

The correlations between differentially expressed lncRNAs and mRNAs were determined. The lncRNA-mRNA co-expression network was established based on the normalized signal intensity of lncRNAs and mRNA. Using Pearson’s correlation coefficient ≥ 0.7,mRNA and encoding genes were determined. Then the lncRNA-mRNA co-expression network was established according to mRNA/lncRNA expression correlation using Cytoscape (The Cytoscape Consortium, San Diego, CA, United States) (Pujana et al., 2007). GO analysis was conducted using Kyoto Encyclopedia of Genes and Genomes (Zhao et al., 2015; Chen R. et al., 2016). GO analysis was also used to determine the biological functions of the adjacent protein-coding genes of the target lncRNAs.

### Statistical Analysis

Statistical analyses were performed using SPSS 20.0 software. The relative expression of target lncRNAs in bone marrow samples from MM and IDA patients was compared by using the Mann-Whitney test. Multiple intergroup comparisons were performed using Kruskal-Wallis H test. Kaplan-Meier survival curve was plotted, and log-rank test was used to detect significant difference in the survival of two groups. The Chi-square test was adopted to analyze the relationship between upregulated and downregulated lncRNAs and chromosomal aberrations. *p* < 0.05 indicated significant difference.

## Results

### Differentially Expressed lncRNAs in the Bone Marrow of MM Patients

**FIGURE 1:**
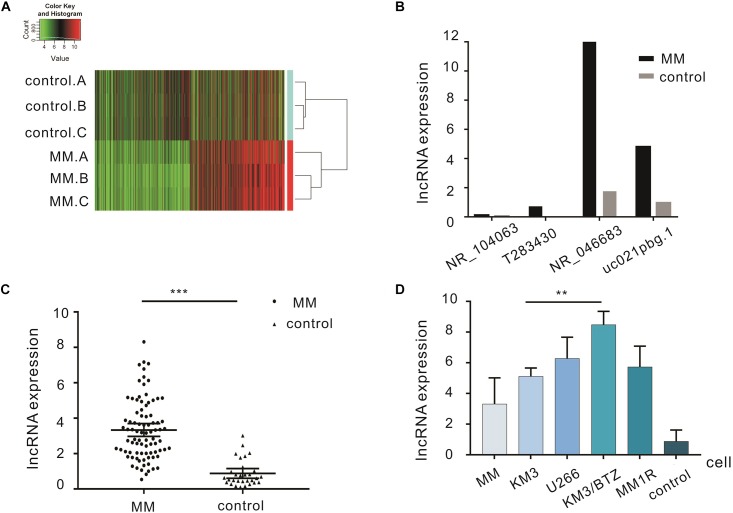
Differentially expressed lncRNAs in the bone marrow of MM patients. **(A)** Cluster analysis diagram of differentially expressed lncRNAs. **(B)** qRT-PCR verification of four lncRNAs in MM patients and controls. **(C)** Expression of lncRNA NR_046683 in the bone marrow of 86 MM patients and 30 controls. **(D)** Expression of lncRNA NR_046683 in MM patients,KM3 cell line, U266 cell line, KM3/BTZ cell line, MM1R cell line and controls. ^∗∗^*p* < 0.005; ^∗∗∗^ *p* < 0.0001. MM, multiple myeloma; qRT-PCR, Real-time quantitative polymerase chain reaction.

From 3 cases of MM and 3 cases of IDA diagnosed preliminarily, high-throughput lncRNA array was used and thousands of differentially expressed lncRNAs were detected. There were 1489 upregulated lncRNAs and 1661 downregulated lncRNAs (Figure 1A). qRT-PCR was performed to verify the 4 most significantly upregulated lncRNAs in 20 MM cases and 10 IDA cases. NR_046683 was most significantly upregulated (Figure 1B), so the expression of lncRNA NR_046683 was further detected in 66 MM patients (Figure 1C). At the same time, we detected its expression in cell lines KM3, U266, KM3/BTZ, and MM1R. The results showed that it was highly expressed in drug-resistant strains (Figure 1D). The results indicated that lncRNA NR_046683 is a reliable biomarker and potential drug target for MM diagnosis.

### Relationship Between lncRNA NR_104063 Expression and Clinicopathological Features of MM Patients

The correlation between lncRNA expression and clinicopathological factors (e.g., age, gender, subtype, and staging) was determined (Table 3). The results showed that lncRNA NR_046683 correlated to the subtype (*H* = 18.2, *p* < 0.001) and ISS staging of MM (*H* = 12.982, *p* = 0.002), but not to age, gender or DS staging.

**Table 3:** Correlations between the relative expression of lncRNA NR_046683 and clinicopathologic features in 86 MM patients.

Clinicopathologic features	Cases	NR_046683 relative expression (Mean ± SD)	U/H value	*P* value
Age (yr)			U = 1120	0.205
<60	48	2.80 ± 1.22		
≥60	38	3.96 ± 2.00		
Sex			U = 602.5	0.174
Male	53	3.60 ± 1.54		
Female	33	2.89 ± 1.83		
Isotype			H = 18.2	0.000*
lgG	43	2.98 ± 1.64		
lgA	22	3.79 ± 1.25		
Light chain	12	4.64 ± 2.00		
Unclassified	9	1.86 ± 0.65		
International staging system			H = 12.982	0.002*
Stage 1	16	2.45 ± 1.65		
Stage 2	33	3.03 ± 1.59		
Stage 3	37	3.98 ± 1.58		
Durie-Salmon stage			H = 1.9	0.387
Stage 1	40	3.26 ± 1.63		
Stage 2	23	2.76 ± 0.61		
Stage 3	26	3.88 ± 2.18		
Cytogenetic abnormality			U = 1154	0.003*
Yes	57	2.66 ± 1.68		
No	29	3.67 ± 1.61		


*SD, standard deviation; U value, Mann-Whitney test; H value, Kruskal-Wallis oxH-test. ^∗^p < 0.05.*

### Clinical Significance of lncRNA Expression of MM Patients

**FIGURE 2:**
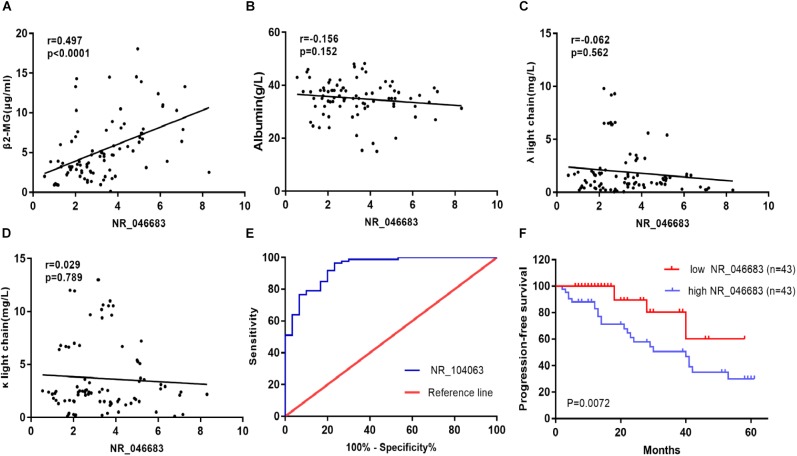
Clinical significance of lncRNA expression in bone marrow of MM patients. **(A)** Correlation between lncRNA expression in bone marrow and serum β_2_M. **(B)** Correlation between lncRNA expression in bone marrow and serum albumin. **(C)** Correlation between lncRNA expression in bone marrow and serum λ light chain. **(D)** Correlation between lncRNA expression in bone marrow and serum κ light chain. **(E)** ROC curve for lncRNA NR_046683, and **(F)** Kaplan-Meier survival curve for low and high expression of lncRNA NR_046683 in MM patients. MM, multiple myeloma; ROC curve, receiver operating characteristic curve; β_2_-MG, β_2_-microglobulin.

Based on the above results, the correlation between lncRNA NR_046683 expression in bone marrow and serum β_2_M, albumin, λ light chain and κ light chain levels was determined in 86 MM patients. The relative expression of NR_046683 correlated positively to β_2_M levels (*r* = 0.497, *p* < 0.001), but not to albumin, λ light chain or κ light chain levels (*r* = -0.156, *p* = 0.152; *r* = -0.062, *p* = 0.562; *r* = 0.029, *p* = 0.789) (Figure 2A–D). The estimated ROC curves were compared between the MM group and control group (Figure 2E), and the sensitivity and specificity on NR_046683 were evaluated. The AUC value was 0.9376 (95%CI 0.8899–0.9853). The correlation between lncRNA and prognosis of MM patients was analyzed (Figure 2F). None of the 86 MM patients dropped out during the follow-up. Using the median expression of NR_046683 in the bone marrows of MM patients, the patients were divided into high and low lncRNA expression groups. The prognostic value of lncRNA for MM was determined based on progression-free survival (PFS).

### Correlation Between lncRNA Expression and Cytogenetic Variation

Using the median expression of lncRNA NR_046683, the patients were divided into low and high expression groups, and the correlation to chromosomal aberrations was assessed using the chi-square test. The over expression of lncRNA NR_046683 was found to be correlated to chromosomal aberrations, such as gain 1q2 (*p* = 0.0096), del. 13q14 (*P* = 0.0288) and *t*(4; 14) (*p* = 0.0266), but not to hyperdiploid (*p* = 0.3722) (Table 4).

**Table 4:** Cytogenetic aberration status distribution between low/high NR_046683 expression groups of MM patients.

Group	Low NR_046683	High NR_046683	*P* value
del (13q14)			*P* = 0.0288^∗^
Positive	30.2% (13/43)	53.5% (23/43)	
Negative	69.8% (30/43)	46.5% (20/43)	
gain 1q21			*P* = 0.0096^∗^
Positive	34.9% (15/43)	62.8% (27/43)	
Negative	65.1% (28/43)	37.2% (16/43)	
*t*(4;14)			*P* = 0.0266^∗^
Positive	9.3% (4/43)	28.9% (12/43)	
Negative	90.7% (39/43)	72.1% (31/43)	
Hyperdiploidy			*P* = 0.3722
Positive	41.9% (18/43)	32.6% (14/43)	
Negative	58.1% (25/43)	67.4% (29/43)	


*MM: multiple myeloma; ^∗^p < 0.05.*

### Functional Analysis of lncRNA

**FIGURE 3:**
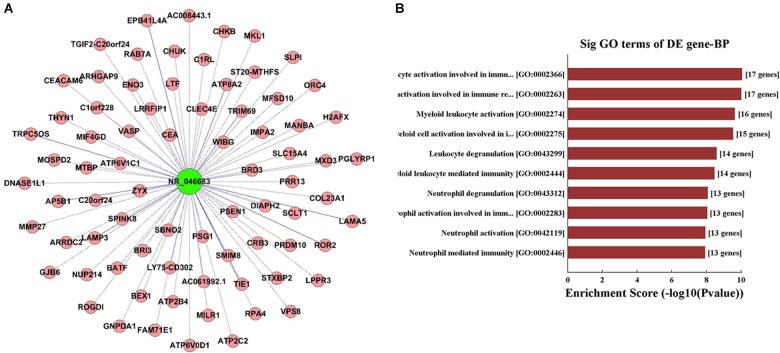
Functional analysis of IncRNA. **(A)** lncRNA-mRNA co-expression network established using lncRNA NR_046683. **(B)** The top ten Enrichment Score value of the significant enrichment terms.

lncRNAs targeted by miRNAs were used for the prediction of target genes and the subsequent functional analysis. lncRNA NR_046683 was selected to establish the lncRNA-mRNA co-expression network (Figure 3A). A total of 76 mRNAs was correlated to lncRNA NR_046683, including ROR2, MTBP and ATP2C2, which are tumor-related protein-coding genes. GO analysis indicated that the genes of lncRNA NR_046683 were mainly involved in the activation of leukocyte activation, bone marrow white blood cells, bone marrow cell activation and immune response (Figure 3B).

## Discussion

MM is a highly heterogenous clonal disease with complex molecular biological characteristics that leads to renal injury, anemia and bone destruction. Along with microenvironmental changes, abnormalities in biomarkers and cytogenetics may already be present before the presentation of symptoms appear (Chen R. et al., 2017; Cowan et al., 2018). Therefore, the search for new indicators for early clinical diagnosis, therapeutic response and prognostic prediction is an urgent issue.

Recent evidences have demonstrated that lncRNA has a key role in the pathogenesis of tumors. lncRNAs can be used for cancer diagnosis, classification, and prognostic evaluation, along with acting as a potential drug target (Huarte, 2015). Previous studies mainly focus on the effect of known lncRNAs on MM. For example, metastasis-associated lung adenocarcinoma transcript 1 (MALAT-1) is overexpressed in some solid tumors (Tripathi et al., 2010; Yang et al., 2013). Cho, S. F. et al. proved the overexpression of MALAT1 of newly diagnosed MM, suggesting that MALAT1 was an eligible biomarker for early progression. lncRNA PDIA3P is found to play an important role in oral squamous cell carcinoma and hepatocellular carcinoma (Kong et al., 2017; Sun et al., 2017). Yang et al. (2018) clarified the potential regulatory mechanism of lncRNA PDIA3P in the pentose phosphate pathway in MM. Moreover, potential targets of metabolic regulation and drug resistance were identified for MM.

For the first time, we combined high-throughput lncRNA array with bioinformatics technique and found an overexpression of lncRNA NR-046683 in bone marrows of MM patients. This result was then verified by using qRT-PCR in large samples (*n* = 86). Combining with the clinicopathological features of MM patients, we found that the NR-046683 expression differed significantly in patients with different MM subtypes and staging. In addition, the overexpression of NR-046683 was closely related to the concentration of β_2_-microglobulin. β_2_-microglobulin has been clinically validated and recognized by most doctors. It is a meaningful indicator closely related to multiple myeloma (Avet-Loiseau et al., 2007). By verifying the relationship between our newly discovered molecules and these indicators, lnc NR-046683 can be used as a biomarker for diagnosis and prognostic prediction in MM.

Chromosomal aberrations are considered relevant to the diagnosis, progression and prognostic prediction of MM (Kumar et al., 2012). In the present study, an overexpression of NR-046683 correlated to the gain 1q21, del. 13q14, and *t*(4; 14), all of which are associated with a poor prognosis of MM (Nemec et al., 2012). To some extent, an overexpression of NR-046683 is correlated to disease progression and predicted poor prognosis. In addition, an analysis of lncRNA-mRNA co-expression indicated that lncRNA is related to ROR2 and MTBP genes (Bi et al., 2015; Debebe and Rathmell, 2015; Lu et al., 2015), which are tumor-associated protein-coding genes. It is implied that lncRNA NR_046683 is possibly involved in the regulation of biological behaviors of tumor cells. Using GO analysis, lncRNA NR_046683-associated mRNA was involved in the activation of bone marrow white blood cells and immune response. This implied that lncRNA NR_046683 may potentially promote MM development and progression by regulating proliferation and apoptosis of bone marrow white blood cells.

The accuracy of bioinformatics technique was verified based on big sample data, indicating that NR-046683 is a candidate target for the treatment of MM. However, the present study is a single-center small-sample-size experiment, from which the results had limitations in guiding clinical treatment (Luo et al., 2018). In the future, the sample size should be enlarged. Understanding the biological features of lncRNA NR-046683 and its involvement in the pathophysiological process of MM is crucial for treatment.

Analysis on lncRNA-mRNA co-expression and GO analysis provides theoretical basis for further investigation. Linkage of lncRNA NR-046683 to mRNA suggests that the lncRNA might provide a potential drug target for small molecule or antibody approaches in controlling the activity of the gene product. As lncRNAs are novel targets with often undefined mechanisms, it is critical to find therapeutic targets for drug discovery during disease progression.

To conclude, lncRNA NR-046683 can serve as a novel biomarker for potential drug target and prognostic prediction in MM.

## Data Availability Statement

The datasets generated for this study can be found in Arraystar Human LncRNA Array V4.0, https://www.arraystar.com/human-lncrna-expression-array-v4-0/.

## Ethics Statement

Ethical approval for this study was obtained from the Ethics Committee of the Third Xiangya Hospital of Central South University (No. 2016121).

## Author Contributions

HD and SJ designed and performed the study. HD, RG, and JL wrote the manuscript with inputs from all authors. YF and YL performed the analytic calculations and statistical analysis. All authors provided critical feedback and helped to shape the research, analysis, and manuscript.

## Conflict of Interest Statement

The authors declare that the research was conducted in the absence of any commercial or financial relationships that could be construed as a potential conflict of interest.
